# Nano-Gradient Materials Prepared by Rotary Swaging

**DOI:** 10.3390/nano11092223

**Published:** 2021-08-29

**Authors:** Qingzhong Mao, Xiang Chen, Jiansheng Li, Yonghao Zhao

**Affiliations:** 1 Nano and Heterogeneous Materials Center, School of Materials Science and Engineering, Nanjing University of Science and Technology, Nanjing 210094, China; 216116000150@njust.edu.cn (Q.M.); lijiansheng@ahpu.edu.cn (J.L.); 2 Anhui Key Laboratory of High-Performance Non-Ferrous Metal Materials, Anhui Polytechnic University, Wuhu 241000, China

**Keywords:** nano-gradient materials, rotary swaging, microstructure, metallic material

## Abstract

Gradient nanostructured metallic materials with a nanostructured surface layer show immense potential for various industrial applications because of their outstanding mechanical, fatigue, corrosion, tribological properties, etc. In the past several decades, various methods for fabricating gradient nanostructure have been developed. Nevertheless, the thickness of gradient microstructure is still in the micrometer scale due to the limitation of preparation techniques. As a traditional but potential technology, rotary swaging (RS) allows gradient stress and strain to be distributed across the radial direction of a bulk cylindrical workpiece. Therefore, in this review paper, we have systematically summarized gradient and even nano-gradient materials prepared by RS. We found that metals processed by RS usually possess inverse nano-gradient, i.e., nano-grains appear in the sample center, texture-gradient and dislocation density-gradient along the radial direction. Moreover, a broad gradient structure is distributed from center to edge of the whole processed rods. In addition, properties including micro-hardness, conductivity, corrosion, etc., of RS processed metals are also reviewed and discussed. Finally, we look forward to the future prospects and further research work for the RS processed materials.

## 1. Introduction

**Figure 1:**
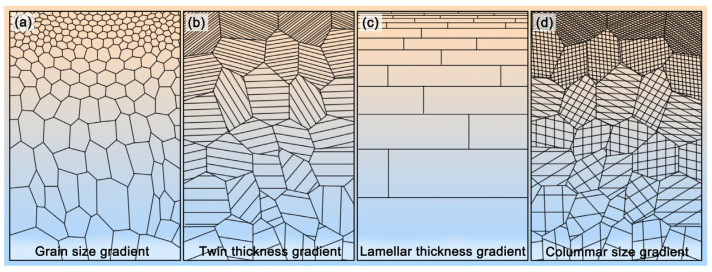
The classification of nano-gradient microstructure with (**a**) grain size gradient, (**b**) twin thickness gradient, (**c**) lamellar thickness gradient and (**d**) columnar size gradient.

Nano-gradient microstructure is commonly defined as a gradient in the internal microstructure from the surface to the interior over a feature length scale, ranging from several nanometers to hundreds of micrometers, or even to millimeters [1,2,3]. As shown in Figure 1, there are four typical gradient nanostructures [4]: grain size gradient—a gradient of grain size from nanometers in the top surface to micrometers in the interior (Figure 1a), twin thickness gradient—a gradient of twin thickness from nanometer to the microscale embedded in grains with uniform size (Figure 1b), lamellar thickness gradient—a gradient of two-dimensional lamellar grains from nanometers to micrometers parallel to the surface (Figure 1c) and columnar size gradient—a gradient of one-dimensional columnar grains from nanometers to micrometers with the same long axis (Figure 1d). As reported by previous literature, at room temperature, the nano-gradient materials often display extraordinary mechanical properties, such as strength–ductility synergy [5,6,7,8], unprecedented strain hardening [9], enhanced fatigue performance [10,11], and remarkable resistance to corrosion [12,13] and wear [14,15,16]. Compared with conventional homogeneous microstructure materials, a significant characteristic of gradient nanostructured materials is that their deformation mechanism is often strongly heterogeneous, occurs progressively and successively, and is accommodated, coordinated and confined by the gradient microstructure [9]. Moreover, the gradient structure often leads to strain and stress gradients and even induces emerging strengthening mechanisms, such as hetero-deformation induced (HDI) strengthening [3]. All of these fascinating results indicate that the gradient nanostructured materials have a broad industrial application and scientific research prospects.

**Figure 2:**
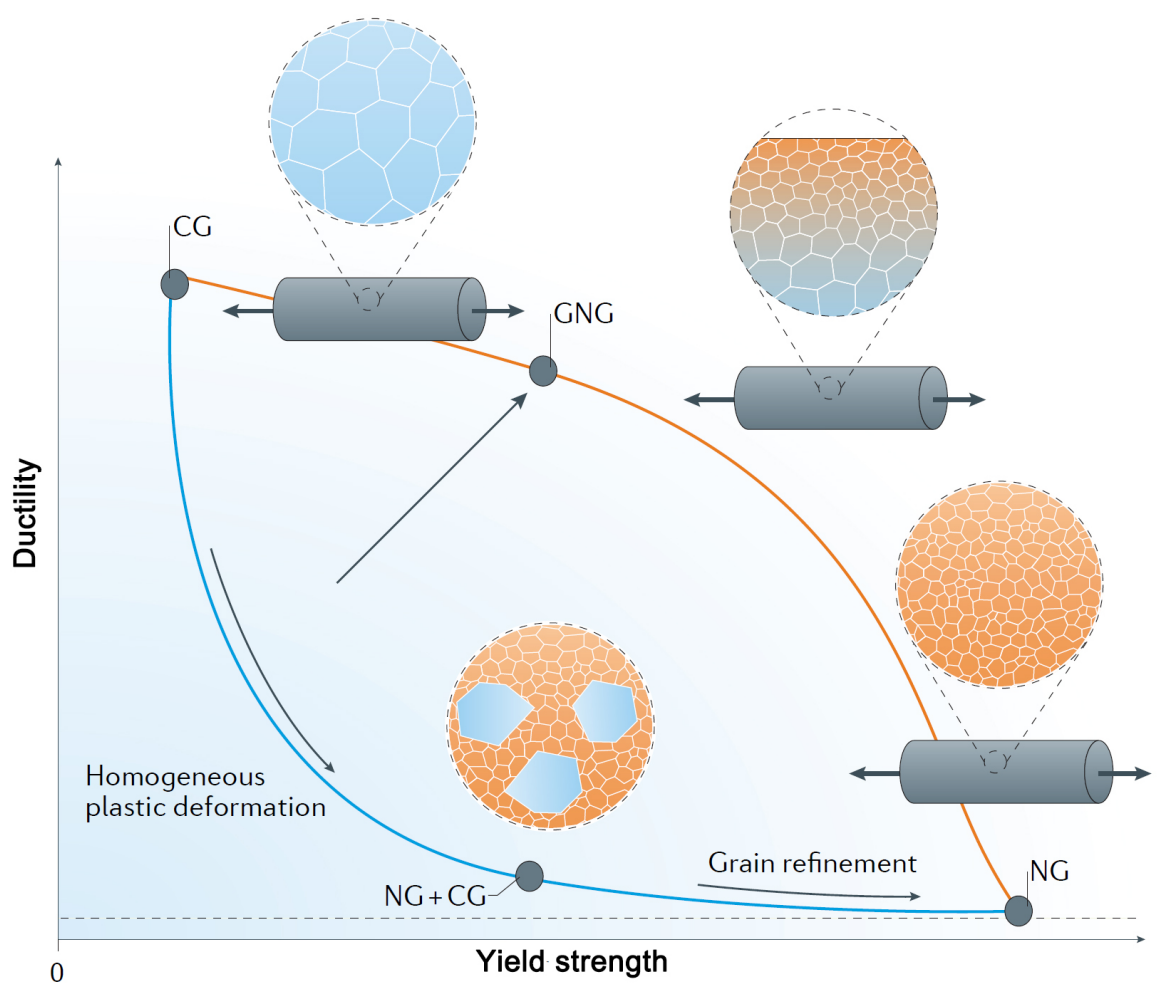
Strength–ductility synergy of gradient nano-grains: CG—coarse grain, GNG—gradient nano-grain, NG—nano-grain. Reproduced with permission from [36]. Copyright Springer Nature, 2016.

In practice, among all four nano-gradient microstructures, only the grain size gradient has been successfully and efficiently prepared by way of surface nanocrystallization, which can be mainly attributed to the gradient strains generated along the depth [17]. Two strategies are implemented to fabricate nano-gradient materials in previous investigations. The first one is laser shock peening (LSP) based on high-energy physics: high-amplitude laser shock waves are generated to impact the surface of the treated materials by absorption of the high-energy laser pulse in an extremely short duration. There exists a gradient microstructure from nanocrystals at the surface to initial coarse-grains at deeper regions [18,19,20]. The second is surface mechanical modification, which is further divided into two cases depending on the contact between the medium and the specimen surface: milling (SMGT—surface mechanical grinding treatment [5], SMRT—surface mechanical rolling treatment [21], FSP—friction stir processing [22], PFSD—platen friction sliding deformation [23], WB—wire brush [24], etc.) and impact (SB—sandblasting [25], RASP—rotary accelerated shot peening [26], SP—shot peening [27], ABSP—air/water blast shot peening [28], USSP—ultrasonic shot peening [29], SFPB—supersonic fine particles bombarding [30], SMAT—surface mechanical attrition treatment [2], SNH—surface nanocrystallization and hardening [31], HESP—high energy shot peening [32], etc.). Table 1 shows the characteristics of gradient structures prepared by four typical techniques. It can be seen that the gradient structure fabricated by LSP has a smaller feature length scale than those structures made by surface mechanical modification. In addition, due to the more energy that can be introduced, the gradient structure formed by the impact of medium has a thicker influence layer compared to the milled surface and can even reach several millimeters in RASP [26]. From Figure 2, we can see that the gradient nano-grained (GNG) structure successfully overcomes the strength–ductility trade-off in metals: nano-grained (NG) metals are strong but brittle, while coarse-grained (CG) metals are weak but ductile [5]. In contrast to the representative ‘banana curve’ (blue curve in Figure 2) for the trade-off between strength and ductility in homogeneously deformed or homogeneous structure metals, the overall strength increase obtained using the gradient nanostructuring is much more marked than the ductility loss [9]. Of course, finer grain size or thicker gradient range may push the strength–ductility curve upwards (orange line in Figure 2). Unfortunately, it should be pointed out that the thickness of the gradient microstructure (usually less than 1 mm) is still in micrometer scale due to the limitation of aforementioned preparation techniques, unavoidably limiting the design of degree and distribution of gradients and, of course, its widespread industrial applications.

As a traditional but potential technology, rotary swaging (RS) allows gradient stress and strain distributed across the whole bulk workpiece. Therefore, in this review, we focus on recent progress in demonstrating the possibility of preparing nano-gradient microstructure throughout bulk materials using RS and the characteristics of nano-gradient microstructure and linked properties.

## 2. Rotary Swaging

### 2.1. Theory of Rotary Swaging

**Figure 3:**
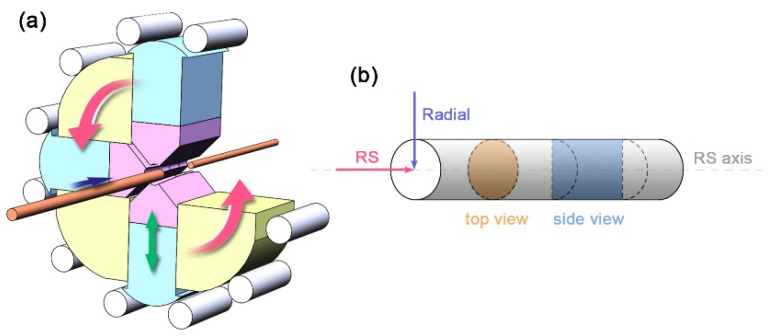
Schematic representation of (**a**) the rotary swaging (RS) deformation of a sample, (**b**) the principal observations of the microstructure.

RS is an incremental forming process utilized to reduce cross sections of bar, tubes, wires and other cylindrical workpieces [37,38,39], which is schematically depicted in Figure 3a. Set of dies (generally two to eight) perform short, high-frequency (from 6800 to 12,000 times per minute), simultaneous radial movements and apply compressive force onto the enclosed workpiece. With every hammering of the die, the workpiece begins to flow and is formed with well precision [39]. Relatively wide strain rate from several to hundreds is controllable by suitable parameters, involving hammer speed, feed speed and the amount of reduction [40,41]. As a net-shape-forming process, the swaged workpiece is obtained with/without only a minimum amount of cutting and processing. In order to measure the deformation of a swaged sample, cross-sectional shrinkage rate (*η*) and true strain (*ε*) are calculated as:

(1) $$\eta =\frac{S_{R}-S_{0}}{S_{0}}$$

(2) $$\varepsilon =\ln\frac{S_{0}}{S_{R}}$$

where *S*_0_ and *S_R_* represent the cross-sectional area of the tube/bar before and after RS, respectively. It should be noted that the actual area of the tube needs to be calculated due to its hollow cross-section. In order to clarify the microstructure of materials, we define the orientations and cross-sections of the workpiece in Figure 3b.

**Figure 4:**
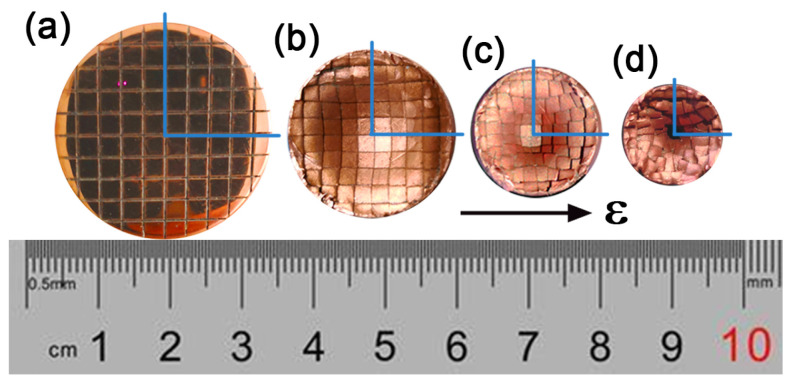
Meshed end faces (top view) of a copper bar before and after RS: (**a**) initial rod, (**b**–**d**) RSed rods, smaller diameter corresponds to larger strain. The blue lines are radials determined by grids, and ε means true strain of RSed material.

RS is deemed as a plasticity enhancement process because of the 2-axial compression and uniaxial tension stress applied to the swaged material, which can effectively refine the grains and boost the mechanical properties of metallic materials. As shown in Figure 4, with high-frequency hammering, the copper rod begins to plastically flow with the reduction in diameter, eventually producing depressions in the two symmetrical end faces. Intuitively, the mesh shrinks along the radial direction without any distortion. Two conclusions are attained: (1) a gradient stress brings about a gradient strain; (2) the strain is radiate outward from low to high along the radial [42]. The presence of residual stresses can significantly affect the mechanical properties of the material. According to the finite element analysis (FEA), the 2-axial compression and uniaxial tension stress of the load also lead to a gradient distribution of residual stress along the radial direction in RS [43,44]. Kunčická et al. reported that RSed tungsten heavy alloy has tensile residual stress exists in the surface and compressive residual stress in the core [43]. However, Singh et al. found the opposite results: compressive stresses at the surface and tensile stresses at the center in Zr-4 alloys processed through RS [44]. The formation of such differences may be related to the nature of the deformed material itself and to the processing parameters of RS (rotation speed, feed speed, number of hammers, etc.). At present, the microstructural gradients are mainly based on surface mechanical treatment (RASP, SMAT, SMGT, etc.), a top-down approach, whereas the medium acts (impact or milling) on the surface of the workpiece to generate a stress and strain gradient, and eventually form a gradient microstructure [9]. Unlike these surface treatment processes, RS is capable of generating a stress/strain gradient across the whole workpiece, and the RSed part also has a wide gradient distribution of residual stresses. Hence, RS, a proven application in industrial production, seems to be a qualified candidate for fabricating bulk nano-gradient materials.

Currently, RS technology is not only used for manufacturing hollow/solid cylindrical shafts, difficult to deform materials and less plastic metals (e.g., tungsten and magnesium alloys), but also for the fabrication of the hollow/solid shafts with variable diameter and of the workpieces with various cross-sections via proper die design [45,46,47]. In addition, because of the significant strengthening effect with low strain [40] and excellent grain refinement ability [48], RS is employed to fabricate the workpieces with ultrafine-grained (UFG) structure or even NG structure.

Recently, RS processed materials were reported to exhibit excellent mechanical [40,41,49,50,51,52,53], fatigue [54,55,56], corrosion [57,58,59,60], tribological [61], electrical properties [61,62,63], etc. We will discuss them in detail in Section 3.

### 2.2. Advantages of Rotary Swaging

Compared with other surface nanocrystallization and severe plastic deformation methods, the RS technique offers several advantages, as follows:

- Most importantly, RS allows gradient stress to be distributed across the bulk workpiece;
- RS has lower tooling cost and higher efficiency in the metal working industry;
- RS is an efficient way to strengthen hexagonal close-packed (HCP) metals by imposing low strain in each pass;
- Applied cooling and heating modes permit RS at expected temperatures;
- Better surface roughness (less than 1 micrometer) and more precise dimension can be achieved via RS;
- The tandem connection of several RS equipment enables the preparation of extra-long materials.

## 3. Gradient Micro-Hardness and Microstructures by Rotary Swaging

### 3.1. Rotary Swaged Face-Centered Cubic Metal

Soft and ductile pure face-centered cubic (FCC), copper and aluminum (Cu and Al) are often chosen to investigate plastic deformation mechanisms. Mao et al. examined the properties (tensile, wear resistance, thermal stability and electrical property) and the microstructure evolution of a copper rod during RS [61], and Gholami et al. investigated the corrosion resistance in Hank’s solution (a simulated body fluid) of them [57].

**Figure 5:**
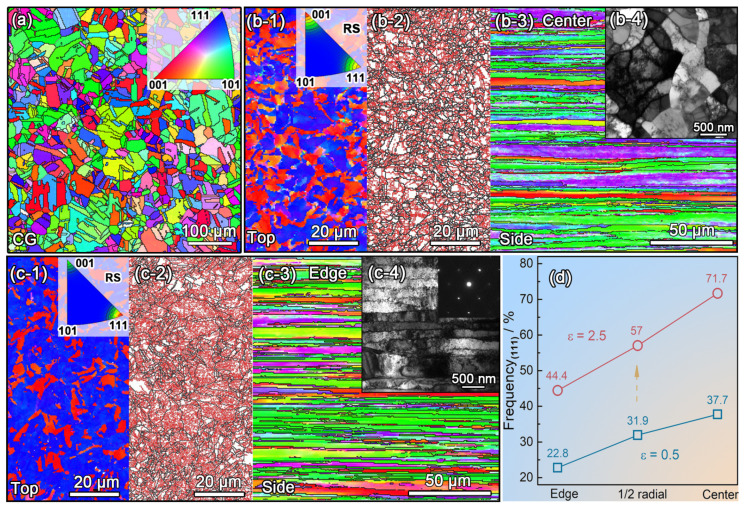
Crystal orientation maps and grain boundary maps of (**a**) initial coarse grains (CG) in copper, (**b1**–**b3**) the center and (**c1**–**c3**) the edge of rod with strain of 2.5, (**b4**) and (**c4**) the center and the edge TEM images of rod with strain of 2.5, (**d**) distribution of (111) texture in RSed rods. Reproduced with permission from [61]. Copyright Springer Nature, 2021.

Figure 5 shows the fractal-like microstructure of RSed Cu with a strain of 2.5 characterized by electron backscattering diffraction (EBSD). Initial equiaxed CG grains (~54 μm) are gradually refined and stretched with an average length of 300 μm and a width of 2 μm (high angle grain boundaries, misorientation larger than 15°) along RS direction, where it contains dislocation cells with a length of 25 μm and a width of 220 nm formed by low angle grain boundaries (misorientation lager than 2° less than 15°), as shown in Figure 5b,c. RS stirs up the otherwise random grain orientations with the material flow; a large number of grains are oriented with <001> and <111> directions parallel to the Cu rod axis, but their proportions are different in the edge and center. As shown in Figure 5d, the RSed rod center has a higher content of <111> texture than edge, and it can be exacerbated by higher strain. According to this phenomenon, RS has the potential to prepare a new gradient structure, texture content gradient—a gradient from several percent to a few tens of percent, unlike the grain size gradient spanning several orders of magnitude. Additionally, compared with the edge (Figure 5(b-2)), the center has a higher density of dislocations (Figure 5(c-2)) i.e., a dislocation density-gradient. Hence, the authors consider the high strength (460 MPa) of RSed Cu is contributed by refined grains, texture and high density of dislocations. Of course, RSed Cu rods have excellent friction properties because it is a natural consequence of high hardness [61].

**Figure 6:**
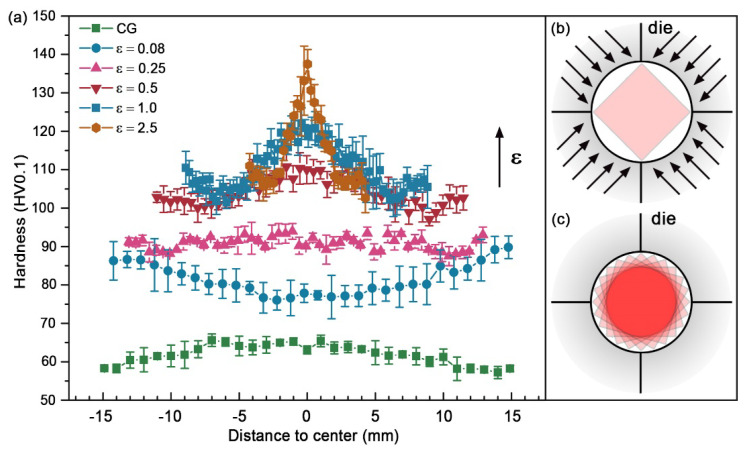
(**a**) Vickers micro-hardness and schematic representation of the RSed copper. (**b**,**c**) schematic of the deformation strain superposition. CG and ε means coarse grains and true strain of RSed material, respectively. Reproduced with permission from [61]. Copyright Springer Nature, 2021.

**Figure 7:**
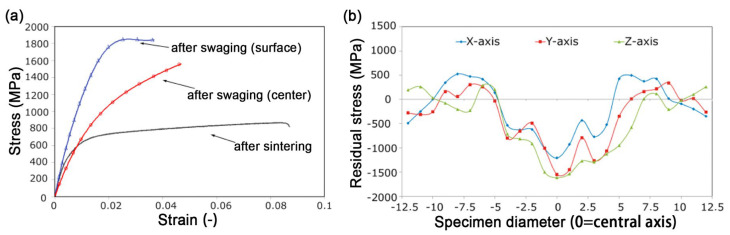
(**a**) Experimental stress–strain curves for sintered and RSed tungsten heavy alloy, (**b**) residual stresses within the RSed tungsten heavy alloy, x and y represent radial directions, z represents RS direction. Reproduced with permission from [43]. Copyright Elsevier, 2017.

Interestingly, according to previous literature, there are three different hardness/strength distributions observed in the cross sections (top view) of RSed rods: (1) V-shaped distribution [40,43]—the strength of edge is higher than that of the center; (2) uniform distribution [64]—the strength of edge is basically equal to that of the center; (3) Λ-shaped [41,50,65]—the strength of edge is lower than that of the center. Fortunately, these hardness distributions are detected in the Cu rods with various strains. Figure 6a shows the curves of Vickers micro-hardness vs. position. At the beginning stage of deformation with a strain of 0.08, the micro-hardness distribution has a V-shape. The higher micro-hardness at the edge is because deformation occurred first at the edge and has not been delivered to the center yet due to (1) the direct contact of the Cu rod surface and swaging dies, (2) the resistance and hysteresis of Cu to the deformation. When strain is larger than 0.5, deformation has been delivered to the Cu rod center, and the micro-hardness distribution changes into a Λ-shape. The highest micro-hardness in the rod center may originate from strain superposition. As schematically shown in Figure 6b,c, every impact from the die does not go around the center, which leads to the maximum strain and micro-hardness in the rod center. The microstructural results also verified the above speculation. The dislocation density at the center is evidently larger than that at the edge when the strain is larger than 2.5. Therefore, the aforementioned three sorts of hardness distributions may be determined by the RS formability of different materials. Moreover, the residual stress also has an effect on strength. Reference [43] point out that the strength distribution is positively correlated to the residual stress, and residual tensile stress is beneficial to the increase strength, as shown in Figure 7. RS can significantly strengthen tungsten alloy with the doubling of tensile strength. However, compared with the center (red line in Figure 7a), the edge has a higher tensile strength (blue line in Figure 7a). Obviously, there is a radially distributed intensity gradient, which coincides with the distribution of residual stresses (Figure 7b).

It is important to note that the RSed microstructure is a recursive fractal structure—long rods containing numerous parallel fibers divided by large angle grain boundaries, which further contains a smaller fibrous network of low angle grain boundaries inside. The concept of macro directional design of the microstructure (MDDM) can fully use the performance of the material by making design according to the specific working direction [62]. For example, the IACS (International Annealed Copper Standard) conductivity of 103% and yield strength above 380 MPa were achieved through this preserved fractal structure prepared by RS and subsequent incomplete annealing. The excellent combination of electrical conductivity and strength is mainly attributed to: (1) the fibrous-like grain boundary network is preserved via incomplete annealing, a large number of grain boundaries parallel to the current direction seduces the scattering of an electron by the grain boundaries; (2) the reduction in dislocation density improve the work hardening of RSed Cu and reduce obstruction of election motion by dislocations. This concept successfully solves the paradox of the strength–electrical conductivity trade-off through clever microstructure design.

In order to investigate the corrosion performance of Cu in the human body, Gholami et al. compared the corrosion resistance property of RSed Cu (UFG structure) with CG Cu in Hank’s solution as the simulated body fluid at 37 °C [57]. According to the potentiodynamic polarization curves, the corrosion potential of Cu rods before and after RS does not change much (~210 ± 10 mV), while their corrosion current density is undulating. The corrosion current density of RSed Cu (strain of 0.5 and 1.0) increased compared with that of the CG Cu counterparts. Further deformation (strain of 2) led to a drop of the corrosion current density. The lowest value of corrosion current density was achieved by RSed Cu with a strain of 3. Fitting the Nyquist plots to the equivalent, the authors found that the RSed Cu (strain of 3) has the maximum resistance (25,883 Ω∙cm^2^). That means the severe RSed Cu with a strain of 3 has the best corrosion resistance compared to CG and low strain conditions. Changes in corrosion properties are related to the evolution of the microstructure during RS: higher corrosion resistance exhibited at high strain is intimately related to a higher residual stress, higher density of (111) planes, finer distribution of grain size and more grain boundaries as a continuous corrosion product on the surface. Accordingly, Abdulstaar et al. studied the corrosion behavior of Al 1050 that was severely deformed by RS via potentiodynamic polarization and a weight loss immersion test in 3.5% NaCl solution at room temperature [59]. The corrosion rate and corrosion current density of RSed Al were significantly lower than those of CG as-received material. After RS2 (with strain of 2) and RS3 (with strain of 3), the corrosion current density dropped to 65% and 75% in comparison to CG Al, and the corrosion rate dropped to 65% after both RS2 and RS3. Observing the surface morphology after the potentiodynamic polarization, the authors found that the formation of a higher number of rectangular shallow deep micro-size metastable pits that have a frequently growth and repassivation, which cover the entire exposed surface, and the pits’ size of RS3 Al is smaller than that of CG Al. Considering that RS is a cumulative strain process and there are obvious residual stresses [43,44,61], it is believed that internal residual stresses play a vital role in inhibiting the dissolution of aluminum, and a larger fraction of grain boundaries and residual stress provide more nucleus to form a dense oxide film.

### 3.2. Rotary Swaged Hexagonal Close-Packed Metals

Magnesium (Mg) and its alloys possess great potential to improve energy efficiency in electronics, automotive and aerospace industries due to its ultra-low density [50,66]. However, as a typical hexagonal close-packed (HCP) metal with insufficient slip systems, Mg and its alloys are intractable to be plastically deformed at room temperature [67]. A plethora of literature reiterated that activating the <c+a> pyramidal slip is an effective method to improve their formability [40,66,67,68].

**Figure 8:**
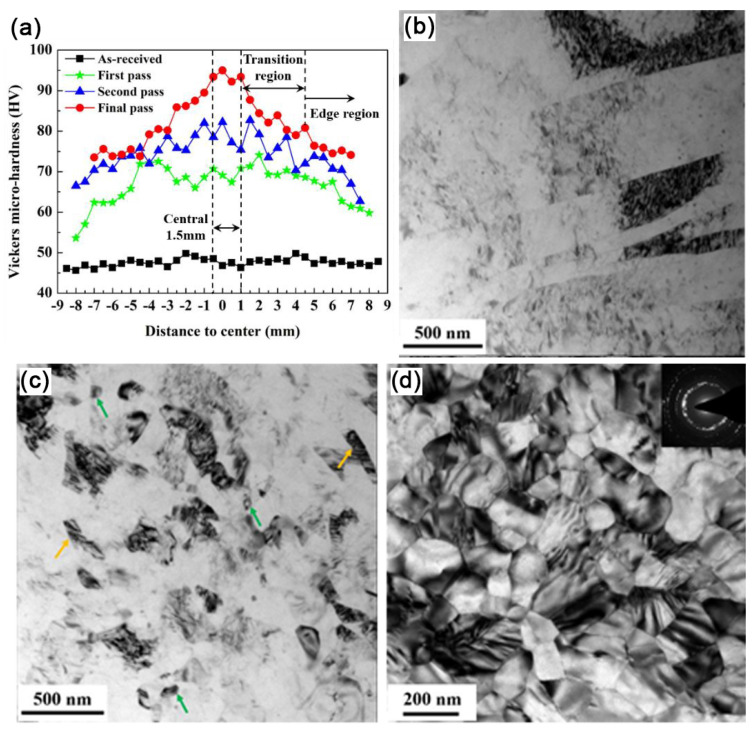
(**a**) Vickers micro-hardness of CG and RSed AZ31B Mg, and TEM images in the center of RSed AZ31B Mg rods (**b**) after one-pass, (**c**) after two-pass and (**d**) after five-pass [69]. Green arrows and yellow arrows represent nano-grains and UFG lamellar twins, respectively. Reproduced with permission from [69]. Copyright Springer Nature, 2021.

**Figure 9:**
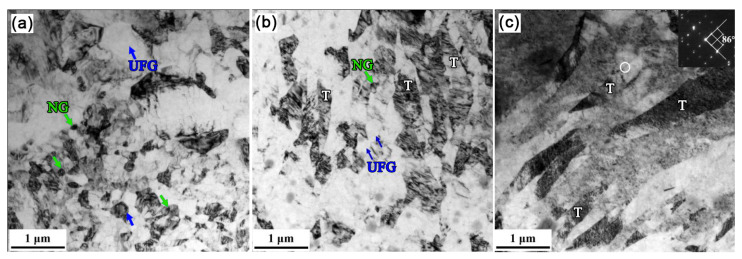
TEM images at different radial positions of RSed AZ31B Mg after five-pass, distance to center of (**a**) 2 mm, (**b**) 4 mm and (**c**) 6.5 mm [69]. NG—nano-grains, UFG—ultrafine grains. (**a**,**b**) shows a transition from NG to UFG, and (**c**) shows a deformation twinning structure near the edge. Reproduced with permission from [69]. Copyright Springer Nature, 2021.

Coincidentally, RS can substantially enhance the plasticity of HCP metals by 2-axial compressive stress. Surprisingly, Wan et al. prepared a bulk nano-gradient AZ31B Mg alloy [69]. As shown in Figure 8a, the swaging process increases the overall micro-hardness, and gradually forms Λ-shape distributions along the radial direction. Moreover, the micro-hardness of the center becomes higher with increasing strain. It can be understood by the microstructure transformation during RS: at the initial stage of grain refinement, dense deformation twins first divided the coarse grains into fine lamellar structures; then a large number of dislocation arrays further refined the twin lamellae into ultrafine grains; finally, randomly orientated nano-grains were formed via dynamic recrystallization as a result of the combined effect of deformation heat and increased stored energy (Figure 8b–d). The inverse nano-gradient microstructure (the grains of 80 nm are placed in the center, and the lamellar grains of thickness of 400 nm are in the edge, Figure 8d and Figure 9) along the rod radial direction was formed by radial hammerings as shown in Figure 6c. The grains in the center were subjected to loading almost equally from all radial directions while those at the edge only experienced loading from one direction. Λ-shaped hardness distribution is found in the cross-section of the RSed AZ31B Mg alloy, mainly due to gradient grain size.

**Figure 10:**
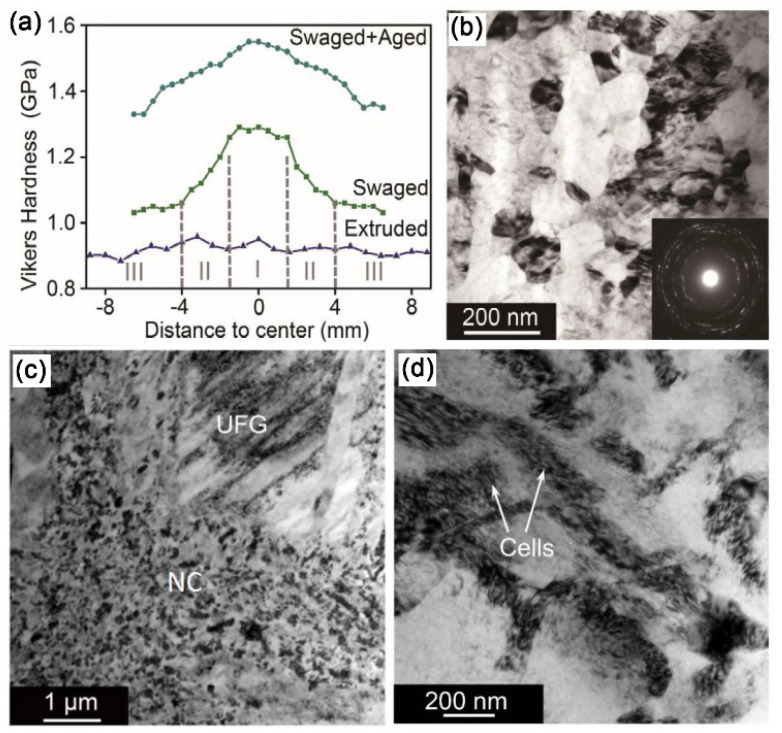
(**a**) Vickers micro-hardness in the top view of the RSed Mg–8Gd–3Y–0.4Zr alloy. TEM image of region (**b**) I, (**c**) II and (**d**) III. Reproduced with permission from [50]. Copyright Elsevier., 2020.

Furthermore, Wan et al. achieved a similar nano-gradient microstructure in Mg–8Gd–3Y–0.4Zr alloy via the same RS process. In the top view of RSed rod, the center has an equiaxed grains of 80 nm (Figure 10b), while dislocation cell structures are formed in the edge (Figure 10d), and ultra-fine grains mixed with nano-sized grains are formed in the transition region from center to edge (Figure 10c). This gradient distribution of grains corresponds to the trend of hardness evolution (Figure 10a).

**Figure 11:**
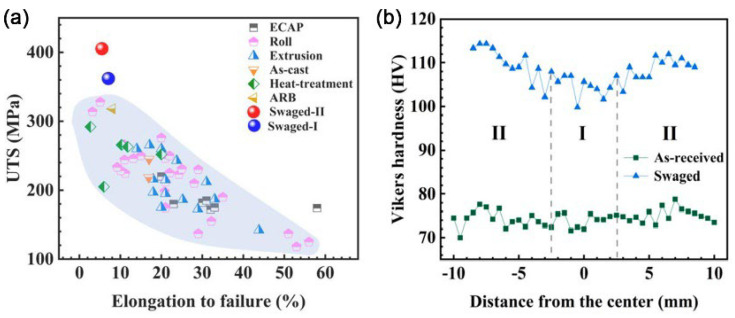
(**a**) Comparison of UTS and elongation to failure with various Mg–Li–X alloys (X: alloying elements) by different SPD techniques in the literature. ECAP—equal channel angular pressing, ARB—accumulative rolling bonding. (**b**) Vickers micro-hardness measured from center to edge along the radial direction of the as-received and swaged Mg–Li alloy rods. Reproduced with permission from [40]. Copyright Taylor & Francis, 2021.

Later on, Yang et al. employed a low-strain RS to fabricate a bulk ultra-light Mg–4Li–3Al–3Zn alloy with new strength record (405 MPa, Figure 10a) [40]. The enhanced mechanical strength is primarily attributable to three reasons: (1) high density of deformation twins; (2) high density of stacking faults; (3) high density of basal <a> and pyramidal <c+a> dislocations. A distinct V-shaped hardness distribution appears in the top view of RSed Mg–4Li–3Al–3Zn alloy (Figure 11b).

Generally, RS can improve the corrosion resistance of the FCC materials [57,58,59,60]. For instance, Bösing et al. investigated the corrosion resistance of RSed stainless steel in phosphate buffered saline (0.2 M NaCl + 0.1 M phosphate buffer solution) and found that a smaller grain size leads to a lower corrosion current density and a higher impedance, pointing to a better resistance against corrosion. However, Minarik et al. investigated the corrosion resistance of the AE42 commercial alloy processed by RS in 0.1 M NaCl solution at room temperature and found that a continuous decrease in the corrosion resistance with an increasing stage of the RS [70]. As the deformation increases, the polarization resistance drops from 165 Ω∙cm^2^ (CG) to 147 Ω∙cm^2^ (RS with a strain of 3). The decrease in the corrosion resistance is attributed to the grain refinement due to the increase in the volume fraction of small grains. Numerous grain boundaries provide diffusion channels for internal atoms to interact with the corrosion environment, and finally, the corrosion resistance is determined by the nature of the corrosion products: the dense layer of oxidation produces leads to increased corrosion resistance, such as Al [59], while flimsy ones accelerate corrosion, such as Mg [70].

**Figure 12:**
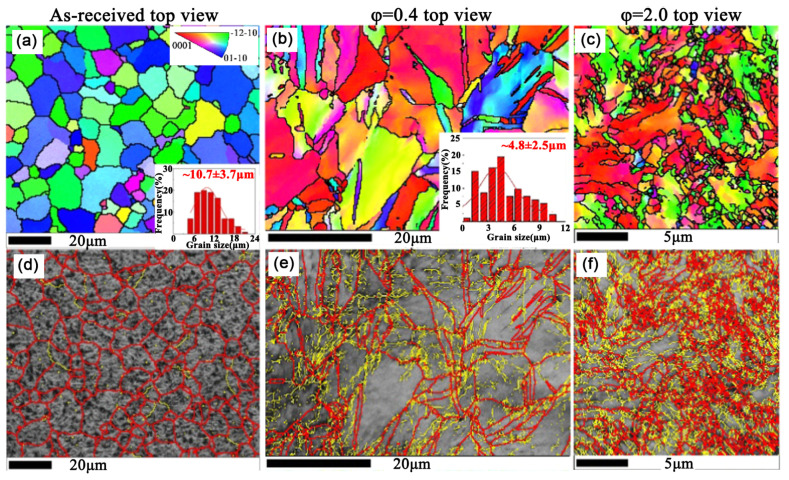
EBSD crystal orientation maps and boundary maps of RSed commercially pure Ti (top view) with a strain of (**a**,**d**) 0; (**b**,**e**) 0.4; (**c**,**f**) 2. Red lines represent misorientation angle > 15°, and yellow lines depict misorientation angle between 2° and 5°. Reproduced with permission from [41]. Copyright Elsevier, 2021.

**Figure 13:**
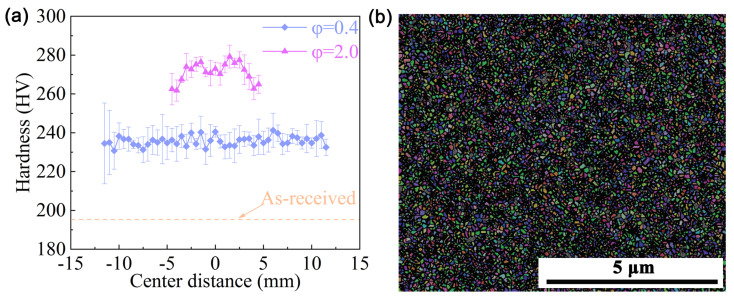
(**a**) Vickers micro-hardness in the top view of the RSed CP Ti rod [41]. Reprinted with permission from ref. [41] Copyright 2021, Elsevier. (**b**) EBSD crystal orientation map of RSed CP Ti with a strain of 2.5. Reproduced with permission from [54]. Copyright Hindawi, 2015.

Meng et al. selected a commercially pure titanium (CP Ti) with a grain size of ~10 micrometer to investigate its microstructure evolution during RS (Figure 12a,d). The deformation changes with the increasing strain, which can be summarized into two processes: (1) at the early stage of RS (strain of 0.4), the {10-12} extension and {11-22} contraction twins occur in the initial coarse grains, as shown in Figure 12b,e, giving rise to the elevation of yield strength and moderate ductility; (2) when the strain is increased to 2 (Figure 12c,f), the twins vanish and are replaced by textured nano-grains with very high strength (~955 MPa). Thus, the high strength of CP Ti is mainly due to the cumulative effect of grain boundary, dislocation and texture strengthening. Moreover, as shown in Figure 13a, in line with aforementioned hardness variation, fickle hardness distribution appears in the RSed Ti. When the strain is low (0.4), the hardness of the center is comparable to that of the edge; as the strain reaches 2, the hardness of the center is significantly higher than that of the edge.

In addition to high strength, Alkhazraji et al. found that RSed CP Ti with a strain of 2.5 also has an excellent fatigue performance at room temperature [54]. CP Ti rod with a grain size of ~100 nm was prepared via the RS process, as shown in Figure 13b, the grain size may have been reduced because a large number of boundaries were not demarcated; however, it is still the finest grains fabricated by RS in Ti and its alloys [37,41,56,71,72,73]. It was shown that the endurance limit stress was dependent on the inverse square root of the grain size. Metals with fine grains show enhanced fatigue property yet result in a high notch sensitivity [54].

**Figure 14:**
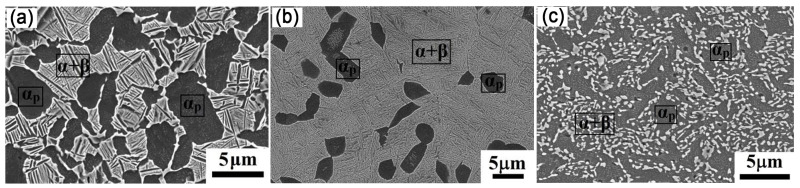
Microstructure of the VT8M-1 alloy: (**a**) in as-received; (**b**) after HT (heat treatment); (**c**) after HT+RS. Reproduced with permission from [51]. Copyright IOP, 2018.

The phase transformations offer a new way of regulating material properties, especially in HCP metals. For example, Ti exists in two allotropic forms: α-Ti is soft, β-Ti is strong, and their mixture has a good strength–ductility combination [74]. Modina et al. investigated the relationship between microstructure and mechanical behavior of a two-phase Ti alloy (VT8M-1) prepared by RS [51,75]. As shown in Figure 14a, the initial microstructure contains ~50% α phase with an average grain size of 5 μm. After heat treatment (940 °C + water quenching + 700 °C/1 h + air cooling), α phase of 2.7 μm was significantly reduced to less than 25% and immersed in α+β phase (Figure 14b). By comparison, the RS significantly refines grain size, and introduces more α phase (Figure 14c). It indicates that the RS may induce phase transformation in Ti alloys. Unfortunately, the mechanism of this phase transformation during RS is unclear.

### 3.3. Rotary Swaged Body Centered Cube Metals

The RS is extensively applied for the processing of pre-sintered and hardly deformed metals, such as ultra-high-strength steel, tungsten (W) alloys and molybdenum (Mo) alloys, due to its incremental character and favorable stress [42,76,77,78]. The W heavy alloys are usually fabricated from powder mixtures consisting of >90 wt% of tungsten powders and elements with lower melting points (Ni, Co, Fe, etc.) usually dissolving between tungsten particles during sintering [42]. The 2-axial compression deformation mechanism enabling the elimination of residual porosity and additional structure refinement.

**Figure 15:**
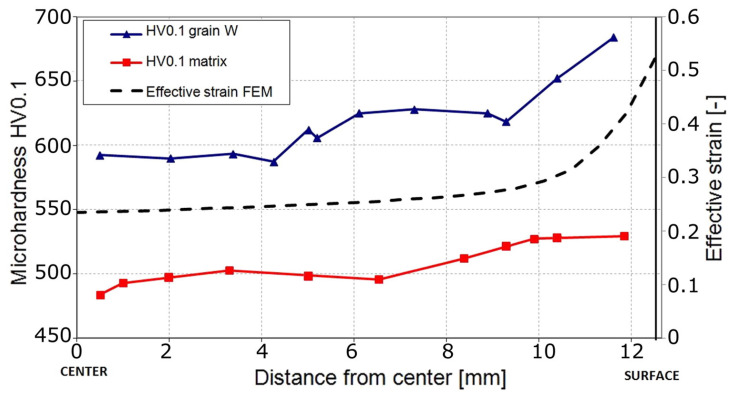
Micro-hardness of W particles and matrix after swaging, including specific regions and comparison to predicted imposed strain. Reproduced with permission from [42]. Copyright Elsevier, 2016.

In order to investigate the effects of cold RS on the structure and properties of a WNiCo heavy alloy, the sintered rod was subjected to a single pass cold RS by Kocich et al. [42]. Figure 15 shows micro-hardness distribution of a cross section of the RSed WNiCo rod, the surface has a higher hardness compared to the center. There is a gradient from the surface to the center, which is consistent with the distribution of the effective strain calculated by the FEM. Higher effective strain result in higher hardness, which also validates the V-shaped distribution of RSed Cu with low strain.

It is well known that increasing the temperature can significantly improve the formability of the metals. Macháčková et al. investigated the deformed behavior of a WNiCo alloy during cold (20 °C) and warm (900 °C) RS, and the effects of the individual processing steps on its structure and properties via numerical prediction and experimental [76]. It was found that compared with cold RS, the warm RS imparted a more homogeneous distribution of the imposed strain due to reduction in strength. However, for both of the RSed rods, the highest strain was observed in their surface regions, which were directly affected by the intensive shear strain introduced by the swaging dies. In addition, both the RSed pieces exhibited the presence of residual stress in the peripheral areas of W agglomerates. However, in HCP metals, the effect of temperature on RS is significant [79]. Estrin et al. demonstrated the viability of the processing of Mg–4.4%Al–0.9%Zn–0.4%Mn alloy prepared by RS with concurrent temperature drops (from 400 down to 200 °C) and found that the decreasing of temperature leads to more strain accumulation, induces secondary deformation twins form within the primary twins and exacerbates the dispersion of textures [79].

## 4. Concluding Remarks and Perspectives

It is surprising that the nano-gradient structure was successfully introduced to various bulk metallic materials with large dimensions by means of an industrial technique of rotary swaging, thanks to gradient stress and strain applied across the radial direction. Moreover, metals processed by RS usually possess an inverse nano-gradient, i.e., nano-grains appear in the sample center, texture-gradient and dislocation density-gradient along the radial direction. In addition, the RS processed metals were proven to have superior properties, including micro-hardness, conductivity, corrosion, etc.

Although the materials prepared by RS ubiquitously display their own specific gradient structures and some intriguing properties, there still exist issues and challenges for the realization of applications. First, more RS processed materials with nano-gradient microstructure need to be revealed in the near future, and the underlying formation mechanisms of the controllable gradient structure should be critically appraised. Second, the fatigue, phase transformation, corrosion and tribological behavior and associated failure mechanisms of the RS processed materials should be systematically examined in an effort to explore its engineering applications. Third, quantitative correspondence between the RS-processed gradient structure and its properties is not yet established, which should spark numerous interests among the materials science community.

## Figures and Tables

**Table 1:** Comparison of methods for fabricating gradient nanostructured metals and alloys.

Fabrication Method	Gradient Distribution in Feature Size (from Top to Interior)	Depth of Nano-Grains	Depth of Gradient	Refs.
SMAT	Tens of nanometers to ~10 μm	~20 μm	~300 μm	[9,17,33]
SMGT	Several nanometers to ~10 μm	~75 μm	~300 μm	[9,34]
LSP	Tens of nanometers to ~100 nm	~50 μm	<1 mm	[9,19]
RASP	Tens of nanometers to ~10 μm	<50 μm	~2 mm	[26,35]

‘Depth of gradient’ is the distance from the nanosized surface to the internal initial structure.

## Data Availability

Data are available from the corresponding author (xiang.chen@njust.edu.cn, yhzhao@njust.edu.cn).
